# Single versus multiple fraction stereotactic radiosurgery for medium-sized brain metastases (4-14 cc in volume): reducing or fractionating the radiosurgery dose?

**DOI:** 10.3389/fonc.2024.1333245

**Published:** 2024-08-13

**Authors:** Philipp Reinhardt, Uzeyir Ahmadli, Emre Uysal, Binaya Kumar Shrestha, Philippe Schucht, Arsany Hakim, Ekin Ermiş

**Affiliations:** ^1^ Department of Radiation Oncology, Inselspital, Bern University Hospital and University of Bern, Bern, Switzerland; ^2^ University Institute of Diagnostic and Interventional Neuroradiology, Inselspital, University Hospital and University of Bern, Bern, Switzerland; ^3^ Department of Radiation Oncology, Prof. Dr. Cemil Tascioglu City Hospital, Istanbul, Türkiye; ^4^ Department of Neurosurgery, Inselspital, Bern University Hospital, University of Bern, Bern, Switzerland

**Keywords:** brain metastases, stereotactic radiosurgery, multiple fraction SRS, single fraction SRS, radionecrosis, MRI, response assessment, recurrence

## Abstract

**Background and purpose:**

Stereotactic radiosurgery (SRS) of brain metastases (BM) and resection cavities is a widely used and effective treatment modality. Based on target lesion size and anatomical location, single fraction SRS (SF-SRS) or multiple fraction SRS (MF-SRS) are applied. Current clinical recommendations conditionally recommend either reduced dose SF-SRS or MF-SRS for medium-sized BM (2–2.9 cm in diameter). Despite excellent local control rates, SRS carries the risk of radionecrosis (RN). The purpose of this study was to assess the 12-months local control (LC) rate and 12-months RN rate of this specific patient population.

**Materials and methods:**

This single-center retrospective study included 54 patients with medium-sized intact BM (n=28) or resection cavities (n=30) treated with either SF-SRS or MF-SRS. Follow-up MRI was used to determine LC and RN using a modification of the “Brain Tumor Reporting and Data System” (BT-RADS) scoring system.

**Results:**

The 12-month LC rate following treatment of intact BM was 66.7% for SF-SRS and 60.0% for MF-SRS (p=1.000). For resection cavities, the 12-month LC rate was 92.9%% after SF-SRS and 46.2% after MF-SRS (p=0.013). For intact BM, RN rate was 17.6% for SF-SRS and 20.0% for MF-SRS (p=1.000). For resection cavities, RN rate was 28.6% for SF-SRS and 20.0% for MF-SRS (p=1.000).

**Conclusion:**

Patients with intact BM showed no statistically significant differences in 12-months LC and RN rate following SF-SRS or MF-SRS. In patients with resection cavities the 12-months LC rate was significantly better following SF-SRS, with no increase in the RNFS.

## Introduction

Brain metastases (BM) commonly occur in solid cancers and are a significant cause of morbidity and mortality (1, 2). Due to the increasing incidence of BM over the past few decades, the true prevalence may be underestimated (3). This increase can be attributed to the growing number of cancer survivors as well as improved identification of BM through the use of modern imaging modalities (3, 4).

Historically, whole brain radiation therapy (WBRT) was the backbone in the treatment of BM (5). Toxicity, especially neuro-cognitive decline after WBRT, prompted investigations of more focal therapies to spare normal brain tissue. Randomized trials showed the safety and efficacy of local radiotherapy, known as stereotactic radiosurgery (SRS) (6–9). With the growing evidence for its usefulness over the past decade, SRS alone has become the standard of care for patients with a good performance status and a limited number of newly diagnosed BM (10).

Despite the excellent local control (LC), especially in small BM treated with single fraction SRS (SF-SRS), physicians must consider the risk of radionecrosis (RN) (11). For large BM (>3 cm in diameter), the benefit of SRS in terms of LC must be weighed against the risk of RN. The Radiation Therapy Oncology Group (RTOG) conducted the phase 1 90-05 trial to estimate the maximum tolerated dose for SF-SRS in previously irradiated patients (12). The authors proposed reducing the radiosurgical dose depending on tumor size and recommended 18 Gy for tumors with diameters of 2–3 cm. In the meantime multi-fraction radiosurgery (MF-SRS) regimes have been introduced (13–15). MF-SRS has been widely utilized as an alternative to reduce the risk of RN. The latest clinical practice guideline from the American Society for Radiation Oncology (ASTRO) recommends that lesions >3 to 4 cm in diameter should be treated with MF-SRS whereas, for small lesions (< 2 cm), SF-SRS is preferred (16). For patients with medium-sized BM (2.0–2.9 cm in diameter) the guideline made no clear recommendation and SF-SRS or MF-SRS is conditionally recommended.

The primary aim of this single-center, retrospective study was to investigate the incidence of local failure (LF) and RN after SF-SRS (1×18 Gy) and MF-SRS (3×8 Gy, 5×6 Gy) in patients with intact BM and resection cavities with target volumes ranging from 4 cm^3^ (2 cm in diameter) to 14 cm^3^ (3 cm in diameter).

## Materials and methods

### Eligibility

This retrospective study was approved by the local ethics committee (Cantonal Ethics Committee Bern, Switzerland, KEK BE 2023-00223). To be eligible, patients had to be treated with SRS between 08/2014 and 01/2022, aged ≥ 18 years, and have histologically confirmed systemic malignancy, with intact BM or resection cavities measuring between 4 cm^3^ (2 cm in diameter) and 14 cm^3^ (3 cm in diameter). Adequate magnetic resonance imaging (MRI) follow-up was also a prerequisite (including at least pre- and post-contrast T1, T2 and diffusion-weighted imaging [DWI]).

### Treatment and dosimetric parameters

A commercial stereotactic mask fixation device was used to immobilize patients in the supine position. Post-contrast enhanced T1- and T2-weighted MRI (1 mm thick) and computed tomography (CT) images (0.75 mm thick) were acquired. CT and MRI scans were registered in the treatment planning system (Accuracy, Precision Treatment Planning) for target volume and normal tissue delineation. Using the post-contrast enhanced T1 sequence and T2 sequence, the gross tumor volume (GTV) for intact metastases was manually delineated. The planning tumor volumes (PTVs) for intact BM were generated by a zero-margin expansion of the GTV. The postoperative rim of enhancement at the edge of the resection cavity and the resection cavity itself were included in the GTV of the resected BM. For resection cavities, the GTV was expanded with a 2 mm margin to the PTV. Surgical tracts and the attached dura was included into the PTV. For patients who received SF-SRS, 1×18 Gy was prescribed. MF-SRS was performed with either 24 Gy in 3 fractions or 30 Gy in 5 fractions. Biologically effective dose (BED) with an α/β of 12 Gy corresponded to 45 Gy for SF-SRS and 40 Gy (3×8 Gy) to 45 Gy (5×6 Gy) for MF-SRS. None of the patients had received previous a WBRT and only 9 had undergone previous SRS targeting a different lesion. Treatment plans were generated using Multiplan treatment planning software version 5.3 or Precision version 1.3 (Accuray. Sunnyvale, CA). The Cyberknife Robotic Radiosurgery System (Accuray, Sunnyvale, CA) was used to deliver the radiation.

To evaluate the risk of RN rates, healthy brain tissue receiving 10 Gy for single-fraction (V10 Gy), 20 Gy for three fractions (V20 Gy) and 30 Gy for five fractions (V30 Gy) were retrospectively generated, using a structure of brain minus PTV. These parameters were not employed during the optimization of the initial treatment plan. Furthermore, a dose gradient index (GI), which quantifies the dose falloff, was retrospectively calculated by using the formula: the volume corresponding to half of the prescription isodose divided by the prescription isodose volume (17). The GI threshold of “3” was used to objectively measure the plan quality (17).

### Follow-up data and radiologic measures

Data were collected during routine clinical procedures (diagnosis, treatment, and follow-up [FU]) and were available via the clinical information system and picture archiving and communication system (PACS) of the Inselspital, Bern University Hospital. All patients had undergone serial MRI every 3–6 months. Our institution’s standardized MRI protocol was followed for imaging acquisition. Images were obtained either on a 1.5T (Magnetom Aera or Avanto, Siemens Healthineers, Erlangen, Germany) or a 3T MR scanner (Magnetom Vida or Skyra, Siemens Healthineers, Erlangen, Germany). However, external MRI exams were also considered for evaluation if the inclusion criteria were fulfilled. The standard brain tumor MRI protocol in our institution included pre- and post-contrast sequences. Pre-contrast sequences include sagittal T1w Sampling Perfection with Application optimized Contrasts using different flip angle Evolution (SPACE), axial fluid-attenuated inversion recovery (FLAIR) and axial DWI. Post-contrast sequences (after intravenous injection of 0.1 mmol/kg gadolinium-based agent) included axial susceptibility weighted imaging (SWI), axial T2w, and sagittal fat-saturated T1 SPACE and coronal fat-saturated FLAIR. DWI was acquired at b values of 0 and 1000 with an automatically calculated apparent diffusion coefficient (ADC) map.

**Figure 1:**
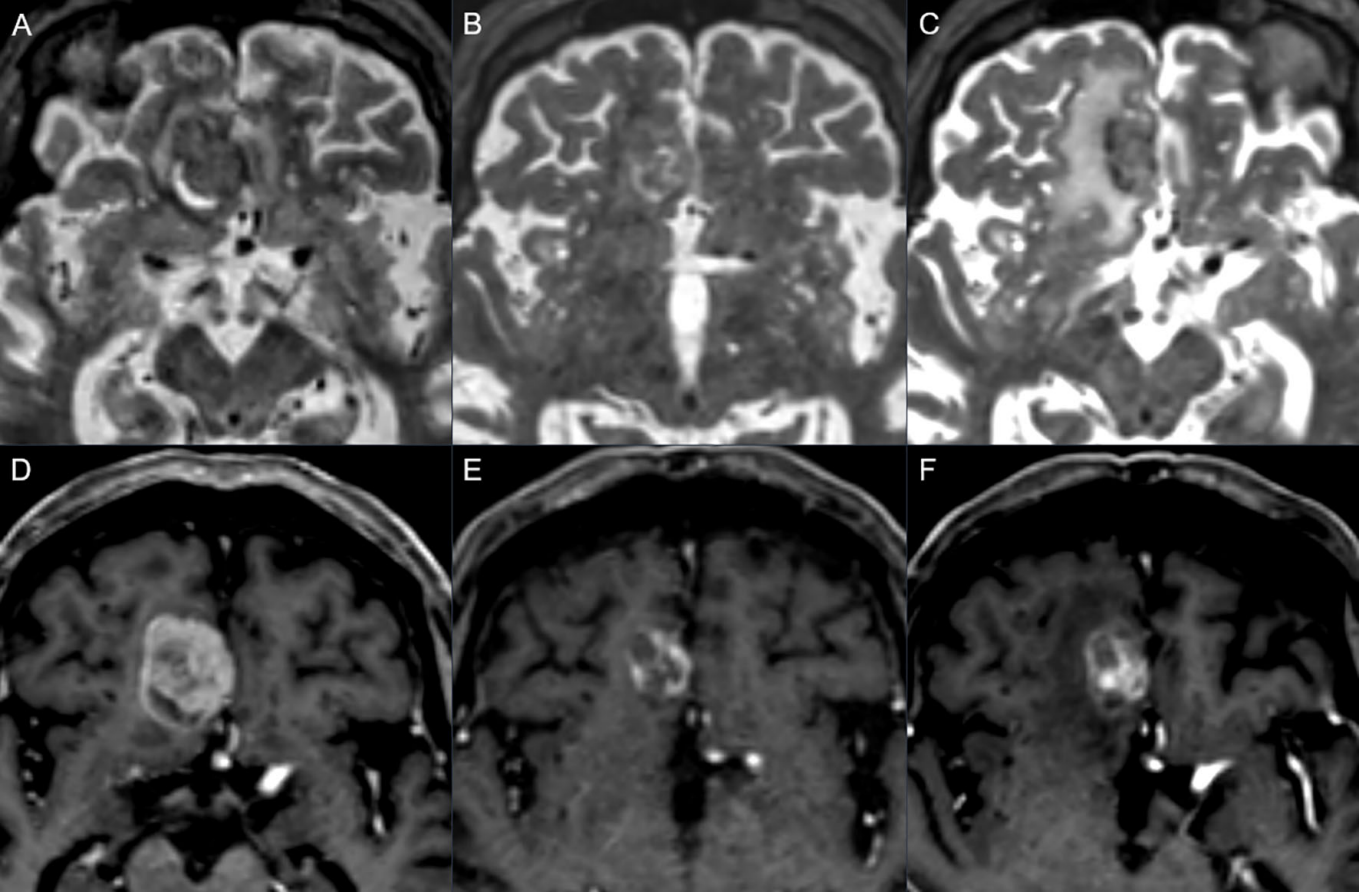
Follow-up example showing progression: axial T2w (upper row) and post-contrast axial T1w (lower row) in a 78-year-old man with metastatic melanoma. Baseline images **(A, D)** show right frontal metastasis, mostly solid with small peripheral cystic changes. Three months after stereotactic radiotherapy **(B, E)** a reduction of the contrast enhancement and the overall diameter was seen with a T1/T2 mismatch, scored as BT-RADS 1. Six months later **(C, F)** there was an increase in the contrast enhancement, representing an increase in the solid part of the lesion with a T1/T2 matching, scored as BT-RADS 3c.

**Figure 2:**
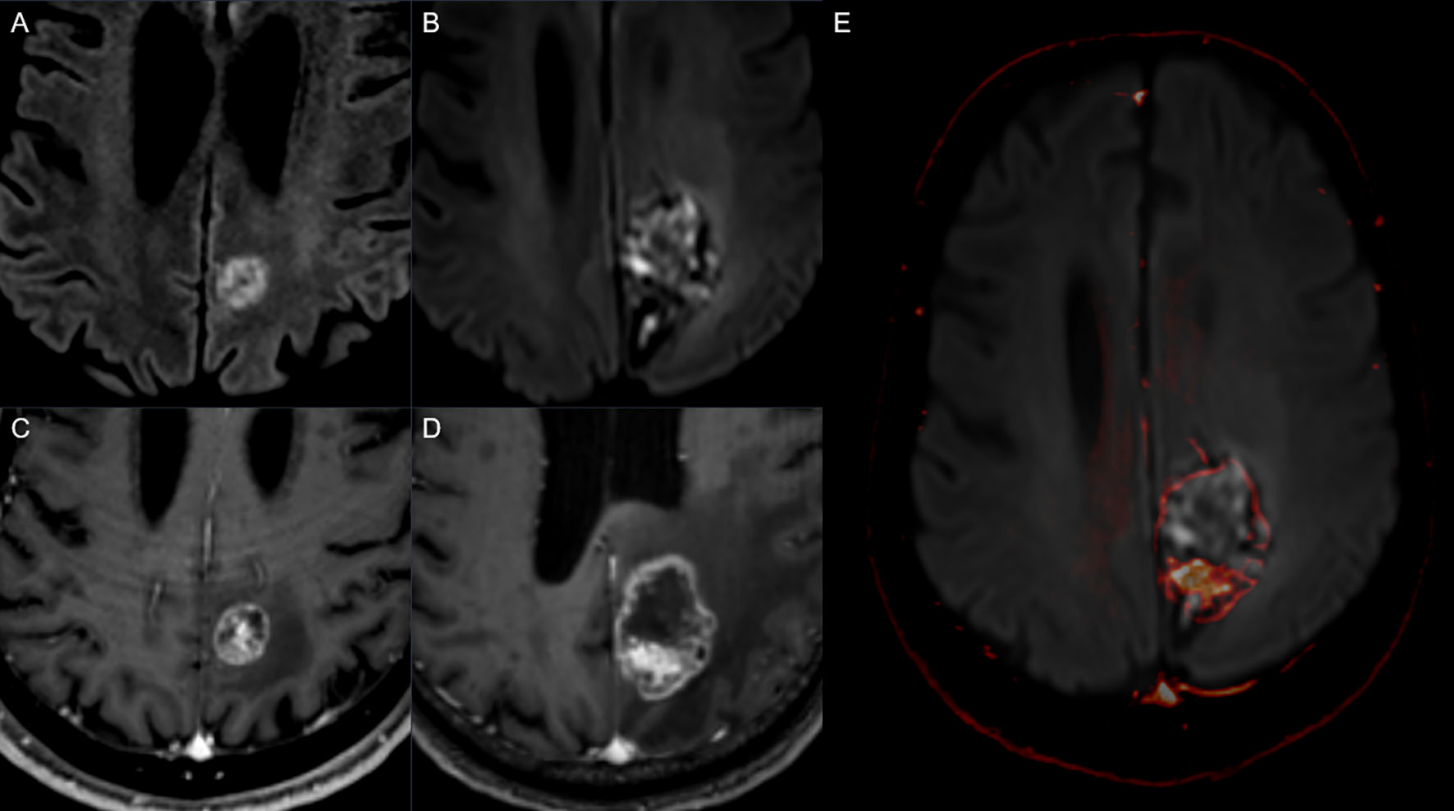
Follow up example showing radionecrosis: Diffusion-weighted MRI sequences (upper row) and post-contrast T1w (lower row) in a 69-year-old man with metastatic non-small cell lung cancer. Baseline imaging **(A, C)** shows left parietal metastasis, mostly solid. Three months after stereotactic radiotherapy **(B, D)** there was an increase in the overall diameter of the lesion with ring enhancement and central diffusion restriction as seen on the fused image **(E)** (post-contrast T1 superimposed on DWI, see arrow). The increase in diameter was attributed to the radionecrosis and scored as BT-RADS 3a.

Single ratings of the images were performed by two board-certified neuroradiologists at baseline and during FU (**Supplementary Table 1**). Baseline imaging was the last MRI before SRS. All lesions were scored using the Brain Tumor Reporting and Data System (BT-RADS) (18). To adapt this classification system for the evaluation of BM, the changes based on T2/FLAIR images without enhancement, which are usually used to evaluate non-enhancing gliomas, were not considered as a marker for progression or response. As the primary endpoint was LF, lesions outside the radiation field were separately evaluated. T1/T2 mismatch (19) and central diffusion restriction (20) were taken into consideration to help in differencing between tumor recurrence and radiation necrosis (**Figures 1**, **2**).

The following were evaluated: change in diameter of the enhancing lesion, new enhancing lesion outside the radiation field, and mass effect. LF or RN was defined depending on the score, (**Supplementary Table 1**). Lesions classified as 3b showed simultaneous signs of LF and RN and could not always be categorized as one or the other. If local salvage treatment was applied to a lesion previously scored as 3b, we defined this as an event in terms of LF without RN. If no local salvage treatment was applied and further MRI FU was performed, this lesion was defined as intermediate 3b with signs of LF and RN.

### Endpoints

The primary endpoint was defined as 12-months LC rate after SRS (defined as time between date of last SRS and suspected LF detected by MRI). Secondary endpoints were RN rate WBRT-free survival (WBRT-FS) and overall survival (OS).

### Statistical analysis

Categorical variables were presented numerically (as a percentage). Continuous variables were reported as median (range). Patient survival was calculated from the time of BM diagnosis and obtained using Kaplan-Meier analysis. For 12-month LC, patients who failed within 12 months and patients who did not fail for at least 12 months were analyzed. Chi-square test or Fisher exact test, where appropriate, were used to compare categorical variables between groups. A p-value of less than 0.05 was deemed statistically significant. Statistical analyses were performed using IBM SPSS Statistics for Windows, version 26 (IBM Corp., Armonk, N.Y., USA).

## Results

### Patient characteristics and treatment

We included 54 patients with 58 BM. Two-thirds of the patients were men, and the median age was 63 years (range 37–89 years). NSCLC (50%) and melanoma (21%) were the most common primary cancers, followed by breast cancer (9%). SRS was performed on 28 intact BM and 30 resection cavities. SF-SRS was administered to 22 of the patients with intact BM, and 6 received MF-SRS. Of the patients with resection cavities, 16 received SF-SRS and 14 MF-SRS. The majority of patients (72.4%) received some form of systemic treatment before, concomitant or after SRS. Among those who had undergone systemic treatment, the most common modalities were chemotherapy (47.4%) and immunotherapy (39.5%). Details of the patient characteristics and treatments are provided in **Table 1**.

**Table 1:** Patient characteristics and treatment .

		All (n=58)	Range or %	Intact BM (n=28)				Resection cavity (n=30)			
				SF-SRS (n=22; 79%)		MF-SRS (n=6; 21%)		SF-SRS (n=16; 53%)		MF-SRS (n=14; 47%)	
Age	Median (range)	63	37–89	63	50–78	58	52–89	62	52–77	66	37–82
Sex	Male	38	65.5	15	68%	3	50%	10	63%	10	71%
	Female	20	34.5	7	32%	3	50%	6	38%	4	29%
KPS	≥90	43	75.4	19	86%	3	50%	13	81%	8	57%
	<90	14	24.6	2	9%	3	50%	3	19%	6	43%
Primary cancer	NSCLC	29	50.0	9	41%	2	33%	13	81%	5	36%
	Melanoma	12	20.7	3	14%	1	17%	2	13%	6	43%
	Breast cancer	5	8.6	1	5%	2	33%	1	6%	1	7%
	Colorectal cancer	4	6.9	2	9%	0	0%	0	0%	2	14%
	Renal cell cancer	3	5.2	3	14%	0	0%	0	0%	0	0%
	Other	5	8.6	4	18%	1	17%	0	0%	0	0%
Status of primary cancer	Controlled	24	41.4	10	45%	3	50%	7	44%	4	29%
	Uncontrolled	13	22.4	5	23%	1	17%	1	6%	6	43%
	Newly diagnosed	21	36.2	7	32%	2	33%	8	50%	4	29%
BM number	Single	28	48.3	7	32%	2	33%	9	56%	10	71%
	Multiple	30	51.7	15	68%	4	67%	7	44%	4	29%
Systemic treatment	Before SRS	24	41,4	12	55%	3	50%	3	19%	6	43%
	After SRS	16	27,6	4	18%	2	33%	9	56%	1	7%
	Concomitant	2	3,4	0	0%	0	0%	0	0%	2	14%
	No	16	27,6	6	27%	1	17%	4	25%	5	36%
Medical therapy	Chemotherapy	18	31,0	7	32%	0	0%	8	50%	3	21%
	Targeted therapy	5	8,6	5	23%	0	0%	0	0%	0	0%
	İmmunotherapy	15	25,9	3	14%	3	50%	3	19%	6	43%
	Hormontherapy	3	5,2	0	0%	2	33%	1	6%	0	0%
	No	16	27,6	6	27%	1	17%	4	25%	5	36%
	missing	1	1,7	1	5%	0	0%	0	0%	0	0%
SF-SRS	1×18 Gy (BED=50,4)	38	65.5	22	100%	0	0%	16	100%	0	0%
MF-SRS	3×8 Gy (BED=43,2)	8	13.8	0	0%	2	33%	0	0%	6	43%
	5×6 Gy (BED=48)	12	20.7	0	0%	4	67%	0	0%	8	57%
Prescribed isodose line	Median (range)	76.5	54.8–81.0	61.50	54,8-80,0	66.00	60,0-75,9	79.5	60,0-80,0	78.7	59,0-81,0
Conformity index	Median (range)	1.18	1.05–1.81	1.18	1.07–1.81	1.20	1.11–1.37	1.12	1.05–1.32	1.14	1.07–1.22
Heterogeneity index	Median (range)	1.29	1.14–1.82	1.50	1.14–1.82	1.51	1.32–1.67	1.26	1.25–1.67	1.26	1.23–1.69
PTV in cc (mean, range)	Median (range)	7.2	4.1–13.1	5.77	4.06–9.53	8.70	4.84–13.05	7.81	5.01–9.40	9.66	5.99–12.64

KPS, Karnofsky Performance Scale; NSCLC, non-small cell lung cancer; BM, brain metastases; SRS, stereotactic radiosurgery; SF-SRS, single fraction stereotactic radiosurgery; MF-SRS, multiple fraction stereotactic radiosurgery.

### Local control intact BM

After a median FU of 21 months, LF occurred in 9 (32.1%) patients. The 12-month LC rate was 65.2%. There was no significant difference between the two fractionation schemes, with a 12-month LC rate of 66.7% for SF-SRS and 60% for MF-SRS (p=1.000) (**Table 2**). A higher LC rate was observed in patients with NSCLC compared to those with other primary tumors (100% vs. 42.9%, p=0.007) (**Supplementary Table 2**). Furthermore, the LC rate of patients with synchronous BM was higher than that of patients with metachronous BM (100% vs. 46.7%, p=0.019). The 12-month LC rate was found to be unaffected by the administered systemic treatment (SF-SRS p=1.000; MF-SRS not applicable) (**Supplementary Table 3**). Furthermore, no significant difference was found according to the type of systemic treatment (SF-SRS p=0.213, MF-SRS p=0.100) (**Supplementary Table 3**). In addition, patients with severe BM symptoms exhibited a significantly lower 12-month LC rate than those with mild or no symptoms (0% vs 85.7% vs 75.0%; p=0.014). A higher prescribed isodose line (IDL) (cutoff IDL >60%) demonstrated a statistically higher 12-month LC rate (p=0.023).

**Table 2:** Overview of results at 12-month follow-up after treatment of intact brain metastases and resection cavities according to fractionation scheme.

	Intact BM (n=28)		p	Cavity (n=30)		p
	SF-SRS (n=22)	MF-SRS (n=6)		SF-SRS (n=16)	MF-SRS (n=14)	
OS	68.2%	83.3%	0.416	87.5%	71.4%	0.845
LC rate	66.7%	60.0%	1.000	92.9%	46.2%	0.013
DBFFS	68.2%	60.0%	0.563	56.3%	64.3%	0.984
DBF rates	36.8%	40.0%	1.000	43.8%	38.5%	0.774
WBRT-FS	90.2%	100%	0.354	68.8%	78.6%	0.578
WBRT rates	12.5%	0%	1.000	31.3%	23.1%	0.697
RN rates	17.6%	20.0%	1.000	28.6%	20.0%	1.000

BM, brain metastases, OS, overall survival, LC, local control, DBFFS, distant brain failure-free survival, DBF, distant brain failure, WBRT-FS, whole brain radiotherapy free survival, WBRT, whole brain radiotherapy, RN, radionecrosis.

### Local control resection cavities

LF occurred in 9 patients after a median FU of 18 months. The 12-month LC rate was 70.4%. The 12-month LC rate was significantly higher in patients undergoing SF-SRS (92.9%) than those treated with MF-SRS (46.2%) (p=0.013) (**Table 2**). No significant difference was found in the administration of systemic treatment (SF-SRS p=0.286; MF-SRS p=0.592) or the type of systemic treatment (SF-SRS p=0.500; MF-SRS p=0.476) (**Supplementary Table 4**). No other factors were associated with significant differences in 12-month LC.

### Radionecrosis

At 6 and 12 months, the RN rates for patients with intact BM were 11.1% and 18.2%, respectively, and 3.4% and 25% for patients with resection cavities. For intact BM, 12-months RN rate was 17.6% for SF-SRS and 20.0% for MF-SRS (p=1.000). For resection cavities, 12-months RN rate was 28.6% for SF-SRS and 20.0% for MF-SRS (p=1.000) (**Table 2**). No difference was found for the 12-month RN rate between SF-SRS and MF-SRS in either group. The treatment planning for intact BM with HI <1.65 (0.0% vs 40%, p=0.029) and IDL >60% (0.0% vs 40.0%, p=0.029) was associated with lower 12-months RN rate. (**Supplementary Table 2**). The results demonstrated no statistically significant difference in 12-month RN rates between a GI of <3 vs >3 (20% vs 25%, p=1.000).

Upon further analyses, the 12-month RN rate was examined for V10 Gy, V20 Gy and V30 Gy in relation to the number of fractions. There was no statistical difference in RN rates for brain minus PTV volume receiving 10 Gy, 20 Gy and 30 Gy for one, three and five fractions with a threshold volume of ≥10cc (21.4% vs 22.2%, p=1.000) (**Supplementary Table 5**).

### Whole brain radiotherapy free survival

Salvage WBRT rates 6 and 12 months after SRS were 3.7% and 9.5% for patients with intact BM, and 23.3% and 27.6% for those with a resected cavity, respectively (**Table 2**). There was no relationship between fractionation and WBRT in either group (**Supplementary Figure 1**).

### Overall survival

OS rates after 6 and 12 months were 96.4% and 71.4% in the patients with intact BM, and 96.7% and 80.0% in those with a resection cavity, respectively. There were no statistically significant differences between fractionation and OS in either group (**Supplementary Figure 2**).

## Discussion

The results of this single-center retrospective study showed no significant difference between SF-SRS and MF-SRS regarding the 12-months LC and 12-months RN in patients with medium-sized intact BM. In patients with resection cavities, however, those who underwent SF-SRS showed a significantly better 12-months LC rate, with no difference in 12-months RN rate. The prescribed dose for SF-SRS is based on the landmark RTOG 90-05 trial, which provides a recommendation for unresected brain metastasis based on the lesion size (12). Following the single-dose regimen of RTOG 90-05, Vogelbaum et al. performed a retrospective study to determine the LC for different intact BM sizes (21). Their results showed a significant benefit in terms of LC of SRS performed with 24 Gy (for BM ≤ 20mm) compared to 18 Gy (for BM 21–30 mm) and 15 Gy (for BM 31–40 mm) (p = 0.0005). LC rates at 1 year in the 24 Gy, 18 Gy and 15 Gy groups were 85%, 49% and 45%, respectively. The authors concluded that LC was proportional to the prescribed dose. The worse LC in patients with larger BM led to the investigation of MF-SRS. Few retrospective studies have evaluated different dose and fractionation regimes in patients with intact brain metastasis. Minniti et al. retrospectively analyzed 289 patients with 343 BM >2 cm in diameter (13). Depending on the size, patients with SF-SRS received either 18 Gy (2–3 cm) or 15–16 Gy (>3 cm). For MF-SRS, 3 × 9 Gy were used, and 53% of the lesions were <3 cm. When compared to BM treated with SF-SRS, lesions treated with MF-SRS showed a significantly higher 1-year LC (91% vs 77%, p=0.01). Additionally, following the administration of MF-SRS, the 1-year incidence of RN was significantly lower (18% vs 9%, p=0.01). These results were confirmed by Chon et al. (22) who analyzed SF-SRS and MF-SRS in patients with BM of 2.5 to 3 cm in diameter. MF-SRS was administered with a median cumulative dose of 35 Gy over 5 fractions, whereas SF-SRS was administered with a median dose of 20 Gy. Both the RN rate after 14 months of FU (29.9% vs 5.3%, p=0.001) and the 1-year LC rate (66.6% vs 92.4%, p=0.028) were significantly better in the MF-SRS-treated group. A meta-analysis comparing SF-SRS and MF-SRS for the definitive and postoperative treatment of BM was published by Lehrer et al. (23). BM were divided into 2 groups based on size (group A: 4–14 cm^3^ or 2–3 cm in diameter; group B: >14 cm^3^ or >3 cm in diameter). For patients with intact BM in group A, the results demonstrated no difference in 1-year LC between SF-SRS and MF-SRS (77.1% vs 92.9%, p=0.18). However, the incidence of RN in this group was considerably reduced following MF-SRS (23.1% vs 7.3%, p=0.003). Furthermore, in patients with resection cavities, the authors found no significant difference in the 1-year LC (only group B was assessed, 62.4% vs 85.7%, p=0.13) between SF-SRS and MF-SRS. The rates of RN were comparable, with no statistically significant difference (7.3% vs 7.5%; p=0.85). A small single-center retrospective study by Donovan et al. (24) looked at RN after SF-SRS (1×24 Gy) and MF-SRS (3×7 Gy). They included 22 patients with 62 BM and a median lesion volume of 0.67ml. There was no difference in the RN rate related to either the maximum dose (OR 1.0, 95% CI: 0.9–1.1), the fractionation scheme (OR 1.0, 95% CI: 0.3–3.6) or a prior WBRT (OR 0.4, 95% CI: 0.2–1.2). However, larger target volumes were associated with an increased risk of RN (OR 3.1, 95% CI: 1.0–9.6).

The dose applied to the 10 cc of healthy brain tissue is a valuable marker for RN. For single-fraction SRS brain volumes receiving 10 Gy and for three and five fraction SRT brain volumes receiving 20Gy and 30 Gy, respectively, have been shown to be predictive for symptomatic necrosis (25–27). In our study, we could not demonstrate any difference in12-month RN rates for the volume cutoff of 10cc with different fractionation schemes.

Our study revealed no difference in OS between SF-SRS and MF-SRS although our results for LFFS and RNFS contradicted those of the earlier studies. A recently published retrospective study by Ostdiek-Wille et al. (28) with a large number of patients, however, supports our results. In their examination of 6961 patients from the National Cancer Database, the median survival times did not differ significantly (10.9 months following SF-SRS and 11.3 months following MF-SRS [p=0.31]).

To our knowledge, no study has so far compared SF-SRS and MF-SRS for treatment of medium-sized resection cavities. We might anticipate an association between radiation dosage and LC for various fractionation schemes, according to a few data from retrospective studies (29–31). However, a recently published summary recommended SF-SRS of higher than 16 Gy or MF-SRS 3 × 8 Gy or > 27.5 Gy in 5 fractions to improve local cavity control (32).

Despite the benefit in terms of LC after postoperative SRS compared to surgery alone, the rate of leptomeningeal disease (LMD) is high and causes significant morbidity without an effective treatment opportunity (33). It is hypothesized that tumor seeding during surgical resection leads to leptomeningeal tumor spread. A few retrospective studies evaluated the efficiency of preoperative SRS in BM (34, 35). Recently, a meta-analysis by Dharnipragada et al. compared pre- and postoperative SRS in BM (36). Both groups were balanced with no significant difference in tumor size distribution. The results demonstrated a significant difference in the rates of local recurrence after one year, with 11% in the preoperative SRS group and 17.5% in the postoperative SRS group (p=0.014). Additionally, the rate of LMD was significantly lower in patients treated with preoperative SRS, with 4.4% vs. 12.3% (p=0.019). No difference was found in terms of RN and OS. Despite these promising results, the optimal fractionation remains undefined. Currently, randomized prospective trials investigating the role of preoperative SRS (37, 38). The results are awaited and could potentially have a significant impact on clinical practice.

The benefits of MF-SRS for treatment of medium-sized intact BM were not supported by the findings of our single-center retrospective study but our study had several limitations. First, due to our inclusion criteria, only a small patient group could be included in this retrospective analysis. Second, the recommendation for prescription of MF-SRS for larger BM has changed in recent years. Data collected in the past revealed a connection between BED and LC. According to Wiggenraad et al. (39), BED_12_ for SRS in intact BM should be at least 40 Gy. Remick et al. (14) also showed an improvement in LC with a BED_10_ ≥ 50 Gy. As stated by Minniti et al. (13) the current recommended scheme for MF-SRS in BM is 27 Gy in 3 fractions. In our investigation, either 30 Gy in 5 fractions (BED_12_ = 45 Gy) or 24 Gy in 3 fractions (BED_12_ = 40 Gy) was used in MF-SRS. The lower BED might be less effective and could lead to a lower LFFS. Third, our groups were not well-balanced, and the treated target volumes in the SF-SRS group were smaller than those in the MF-SRS group. Fourth, it is difficult to compare studies since there is no definition of a medium-sized BM. Most studies assessed the BM size based on the diameter on axial MRI slices. Additionally, target volumes were substantially larger when an extra GTV to PTV margin was applied. In our opinion, only perfectly spherical lesions would be appropriate for this 2D assessment. We therefore used a 3D measurement to determine the BM size in accordance with the GTV. In contrast, no margin was provided to expand the GTV to the PTV in intact BM. For future research, a consistent definition of “medium-sized” BM is needed. Furthermore, the differentiation of LF and RN is challenging. Without a histological confirmation, the MRI-based findings could be misleading. According to our modification of the BT-RADS scoring system, lesions that scored 3b simultaneously showed characteristics of LF and RN. Therefore, the incidence of LC and RN in our analysis could have been overestimated. Additionally, there is a known limitation of using only conventional imaging in the differentiation between tumor and necrosis. The inclusion of advanced imaging could potentially be beneficial to distinguish RN from LF but was not feasible in our study.

Overall, there is so far no evidence from prospective trials evaluating SF- and MF-SRS in patients with medium-sized intact BM and resection cavities. Currently, two prospective trials are recruiting patients to answer this question (NCT05160818, NCT03697343).

## Conclusion

Our results showed no difference in LC or RN following treatment with SF-SRS and MF-SRS for intact BM. In patients with resection cavities, SF-SRS resulted in significantly better LC, without increasing RNFS.

## Data Availability

The original contributions presented in the study are included in the article/**Supplementary Material**. Further inquiries can be directed to the corresponding author.
