# Supplementary material for: Single versus multiple fraction stereotactic radiosurgery for medium-sized brain metastases (4-14 cc in volume): reducing or fractionating the radiosurgery dose?
Source: Front Oncol. 2024 Aug 13;14:1333245. doi: 10.3389/fonc.2024.1333245 (PMC11347337; doi:10.3389/fonc.2024.1333245)
Supplement: Supplementary file 1 [file DataSheet1.docx]

**Supplementary material**

| **Score** | **Title** | **Subtitle** | **Description** | **LF** | **RN** |
| --- | --- | --- | --- | --- | --- |
| 0 | Not scored |  | Baseline |  |  |
| 1 | Imaging improvement | Improvement | Improvement seen on imaging reflect decreasing tumor burden and/or improving treatment | No | No |
| 2 | No change |  | No appreciable change from previously | No | No |
| 3 | Imaging worsening | 3a: Favors treatment effect | Worsening findings seen on imaging favoring treatment effects | No | Yes |
|  |  | 3b: Indeterminate | Worsening findings seen on imaging favoring an indeterminate mix of treatment effect and tumor | Yes | Yes |
|  |  | 3c: Favors tumor progression | Worsening findings seen on imaging favoring increasing tumor burden | Yes | No |
| 4 | Imaging worsening |  | Worsening of findings seen on imaging highly suspicious for tumor progression | Yes | No |
| Abbreviations: LF: local failure; RN: radionecrosis | | | | | |

Supplementary material 1 - BT-RADS classification modified from Weinberg et al. [1] to score local response of metastasis. N.B.: new lesions outside the radiation field were scored separately

|  | **Intact BM (n=23)** | | **Resection cavity (n=27)** | |
| --- | --- | --- | --- | --- |
| **Groups** | **12-month LC rate** | **p** | **12-month LC rate** | **p** |
| All patients | 65.2% |  | 70.4% |  |
| Fractionation | | 1.000 |  | 0.013 |
| Single | 66.7% |  | 92.9% |  |
| Multiple | 60.0% |  | 46.2% |  |
| Primary diagnosis | | 0.007 |  | 0.102 |
| NSCLC | 100.0% |  | 82.4% |  |
| Others | 42.9% |  | 50.0% |  |
| Time of BM | | 0.019 |  | 0.696 |
| Synchronous | 100.0% |  | 75.0 |  |
| Metachronous | 46.7% |  | 66.7 |  |
| Symptomatic brain metastasis | | 0.014 |  | 0.608 |
| No | 75.0% |  | 78.6% |  |
| Few symptoms | 85.7% |  | 60.0% |  |
| Severe symptoms | 0.0% |  | 66.7% |  |
| Target volume | | 0.179 |  | 1.000 |
| <6.6 cc | 78.6% |  | 75.0% |  |
| ≥6.6 cc | 44.4% |  | 69.6% |  |
| Isodose line | | 0.023 |  | 1.000 |
| ≤60% | 33.3% |  | 100.0% |  |
| >60% | 85.7% |  | 68.0% |  |
| Systemtic treatment | | 1.000 |  | 1.000 |
| Yes | 65.0% |  | 72.2% |  |
| No | 66.7% |  | 66.7% |  |
| Time of systemtic treatment | | 0.175 |  | 0.428 |
| Before SRS | 53.3% |  | 57.1% |  |
| After SRS | 100.0% |  | 88.9% |  |
| Concurrent | - |  | 50.0% |  |
| No | 66.7% |  | 66.7% |  |
| Type of systemtic treatment | | 0.166 |  | 0.158 |
| Chemotherapy | 50.0% |  | 90.0% |  |
| Targeted | 100.0% |  | - |  |
| Immunotherapy | 66.7% |  | 50.0% |  |
| Hormontherapy | 0.0% |  | - |  |
| No | 66.7% |  | 66.7% |  |
|  | **12-month RN rate, n=22** | **p** | **12-month RN rate, n=24** | **p** |
| All patients | 18.2% |  | 25.0% |  |
| Isodose line | | 0.029 |  | 1.000 |
| ≤60% | 40.0% |  | 27.3% |  |
| >60% | 0.0% |  | 25.0% |  |
| Homogeneity index | | 0.029 |  | NA |
| <1.65 | 0.0% |  |  |  |
| ≥1.65 | 40.0% |  |  |  |

Supplementary material 2 - Overview results for intact brain metastases and resection cavities

**Intact BM (n=23)**

|  | **SF-SRS (n=18)** | | **MF-SRS (n=5)** | | |
| --- | --- | --- | --- | --- | --- |
| **Groups** | **12-month LC rate** | **p** | **12-month LC rate** | **p** |  |
| Systemic treatment | | 1.000 |  | - |  |
| Yes | 66.7% |  | 60.0% |  |  |
| No | 66.7% |  | - |  |  |
| Time of systemic treatment | | 0.760 |  | 0.400 | |
| Before SRS | 58.3% |  | 33.3% |  | |
| After SRS | 100.0% |  | 100.0% |  | |
| Concurrent | - |  | - |  | |
| No | 66.7% |  | - |  | |
| Type of systemic treatment | | 0.213 |  | 0.100 | |
| Chemotherapy | 50.0% |  | - |  | |
| Targeted therapy | 100.0% |  | - |  | |
| Immunotherapy | 33.3% |  | 100.0% |  | |
| Hormontherapy | - |  | 0.0% |  | |
| No | 66.7% |  | - |  | |

Supplementary material 3 – Association of systemtic treatment and 12-months LC rate in patients with intact BM

**Resection cavity (n=27)**

|  | **SF-SRS (n=14)** | | **MS-SRS (n=13)** | | |
| --- | --- | --- | --- | --- | --- |
| **Groups** | **12-month LC rate** | **p** | **12-month LC rate** | **p** |  |
| Systemic treatment | | 0.286 |  | 0.592 |  |
| Yes | 100% |  | 37.5% |  |  |
| No | 75% |  | 60.0% |  |  |
| Time of systemic treatment | | 0.429 |  | 1.000 | |
| Before SRS | 100.0% |  | 40.0% |  | |
| After SRS | 100.0% |  | - |  | |
| Concurrent | - |  | 50.0% |  | |
| No | 75.0% |  | 60.0% |  | |
| Type of systemic treatment | | 0.500 |  | 0.476 | |
| Chemotherapy | 100.0% |  | 66.7% |  | |
| Targeted therapy | - |  | - |  | |
| Immunotherapy | 100.0% |  | 20.0% |  | |
| Hormontherapy | - |  | - |  | |
| No | 75.0% |  | 60.0% |  | |

Supplementary material 4 – Association of systemtic treatment and 12-months LC rate in patients with resection cavities

|  |  | 12-months RN rate | |
| --- | --- | --- | --- |
|  |  | yes | no |
| V10 Gy, V20 Gy or V30 Gy | <10cc | 22 (78.6%) | 6 (21.4%) |
|  | >10cc | 7 (77.8%) | 2 (22.2%) |
| Gradient index | <3 | 20 (80%) | 5 (20%) |
|  | >3 | 9 (75%) | 3 (25%) |

Supplementary material 5 – Further evaluation of 12-months RN rate. V10 Gy, V20 Gy or V30 Gy: brain minus PTV volume receiving 10 Gy, 20 Gy and 30 Gy for one, three and five fractions. Gradient index: volume corresponding to half of the prescription isodose divided by the prescription isodose volume.


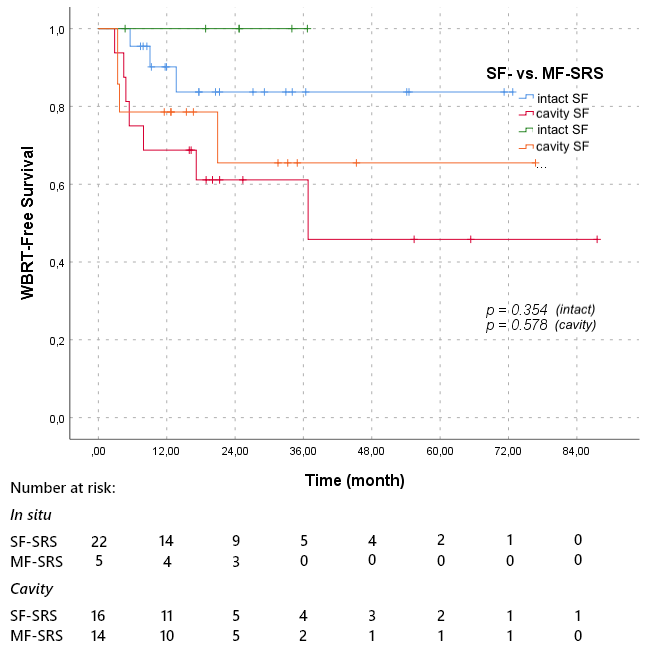
 Supplementary material 6 - Whole brain radiotherapy (WBRT)-free survival


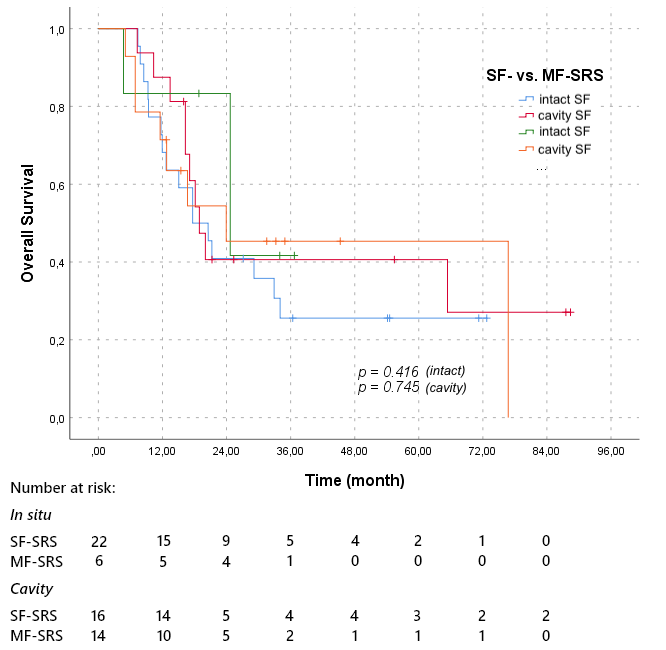
 Supplementary material 7 - Overall survival
